# Analysis of Extracellular RNA by Digital PCR

**DOI:** 10.3389/fonc.2014.00129

**Published:** 2014-06-04

**Authors:** Kenji Takahashi, Irene K. Yan, Chaeyoung Kim, Jungsu Kim, Tushar Patel

**Affiliations:** ^1^Department of Transplantation and Cancer Biology, Mayo Clinic, Jacksonville, FL, USA; ^2^Department of Neuroscience, Mayo Clinic, Jacksonville, FL, USA

**Keywords:** non-coding RNA, exosomes, extracellular vesicles, gene expression, detection, real-time PCR

## Abstract

The transfer of extracellular RNA is emerging as an important mechanism for inter-cellular communication. The ability for the transfer of functionally active RNA molecules from one cell to another within vesicles such as exosomes enables a cell to modulate cellular signaling and biological processes within recipient cells. The study of extracellular RNA requires sensitive methods for the detection of these molecules. In this methods article, we will describe protocols for the detection of such extracellular RNA using sensitive detection technologies such as digital PCR. These protocols should be valuable to researchers interested in the role and contribution of extracellular RNA to tumor cell biology.

## Introduction

An emerging mechanism for cell-to-cell communication involves the transfer of biologically active RNA molecules released into the extracellular space and that can be taken up by recipient cells. The potential importance of this mechanism is highlighted by the demonstration of biological effects in recipient cells both within the local environment as well as at a distance. Several different types of RNA may be released, and include both protein-coding RNA as well as non-coding RNA. The latter are of particular interest because modulation of gene expression is a key feature of non-coding RNA such as the microRNA.

The isolation and characterization of extracellular RNA thus is a necessary tool for understanding biological processes involving inter-cellular communication. Extracellular RNA can be released from cells as native RNAs, associated with proteins, or enclosed within extracellular vesicles (EV) such as exosomes. Extracellular RNA can be detected within EV in the circulation. Moreover, it has been reported that microRNAs that are not enclosed within EV can be isolated from the circulation (1–3). Extracellular RNA is susceptible to degradation by circulating RNAses (2). However, RNA enclosed within EV may be protected from such degradation. These EV differ in their biogenesis. They include exosomes, microvesicles, and apoptotic bodies. In addition to RNA such as mRNA or non-coding RNA, these vesicles may also contain proteins, and lipids. They are released from a wide variety of normal or diseased cells (4, 5). The content and postulated biological roles of these EV may vary with the cells of origin (5, 6). Recent studies have confirmed that EV can transfer RNAs such as ncRNAs from one cell to another and thereby contribute to important roles in inter-cellular communication (3–9). We describe methods for the isolation of EV obtained from human serum or from cells in culture, and for the extracting extracellular RNA from these EV.

Detection of RNA has relied on the use of quantitative real-time polymerase chain reaction (qPCR). Although well-established, and robust, the use of qPCR is limited by the sensitivity in detecting the small amounts of RNA that are obtained from conventional preparations of extracellular RNA. Digital polymerase chain reaction (dPCR) provides an alternative approach for the detection of gene expression in the setting where the amount of target RNA is low and approaching the limits of sensitivity of qPCR (10). Recent studies have compared the two technologies and reported improved precision and reproducibility with dPCR (11). dPCR offers the ability to obtain absolute quantification of gene expression, thereby avoiding reliance on the need for invariant genes (which may not be possible in the analysis of extracellular RNA). In addition, detection using dPCR is more tolerant to PCR inhibitors because it is based on the detection of presence or absence of a reaction end-point.

Digital PCR involves the partitioning of a sample into multiple separate reactions that result in several thousands or millions of individual reactions. This can be accomplished through the use of microfluidic chips or by generating microdroplets. With droplet digital PCR (ddPCR), partitioning occurs within nanoliter or picoliter sized droplets. The reaction undergoes end-point PCR, and droplets will contain some copies or no copies of target sequence of interest. These droplets are individually analyzed using a fluorescence detector (e.g., QX100, Bio-Rad), or flow cytometry (RainDrop, RainDance). Positive droplets are counted and used to estimate the target concentration. The true concentration may be underestimated because a positive droplet cannot differentiate between the number of molecules present in each partition. This can be addressed by using the Poisson equation to calculate the concentration based on the number of negative droplets and using the following formula: copies per droplet = −*ln*(1 − *p*) where *p* = fraction of positive droplets. Increasing the number of partitions offers the potential to increase sensitivity for the detection of very small amounts of targets, and can be accomplished through microfluidic based PCR platforms such as the BioMark (Fluidigm) and Open Array (Life Technologies). Increasingly, dPCR is being used for various applications such as absolute quantification, rare event detection, and copy number variation. Therefore, the use of dPCR could be a very useful tool for the detection and quantitation of extracellular RNA. We report studies that illustrate the use of dPCR for the detection of extracellular RNA.

## Materials and Methods

### Isolation of extracellular vesicles

We describe herein an approach involving the use of sequential centrifugation for the isolation of EV as an example. This approach is based on previous reports from our laboratory and others (1, 5, 12, 13) and has been adapted for isolation of EV from cells in culture or from serum. Isolation of specific types of EV such as exosomes would require additional procedures. There are several other approaches that have been used for the isolation of EV, such as the use of affinity filtration, precipitation, or affinity-based isolation techniques. Several commercial products based on these approaches are available. Protocols using these approaches vary with the type of biological sample that is being analyzed. It should be noted that the EV preparations obtained vary with the approach used for isolation and depending on the isolation approach used, they may contain non-vesicular RNA or restricted EV populations.

For cells in culture, cells are cultured in media that is pre-depleted of EV by centrifugation as follows: cell culture medium is centrifuged at 100,000 × *g* at 4°C overnight. The supernatant is then filtered through a 0.22-μm filter and stored at 4°C. Cells are cultured in EV depleted culture medium for 3–4 days. For most isolations, we have collected EV from supernatants obtained from at least 16 10-cm culture dishes in order to obtain a sufficient yield for downstream studies. For isolation of EV from serum, 500 μl of serum is first diluted 1:3 with cold phosphate buffered saline (PBS).

Isolation of EV is then performed by sequential centrifugations that result in removal of cells, removal of cell debris, and larger vesicles, and ultracentrifugation to generate a residua of EV. The samples are first centrifuged at 300 × *g* for 10 min, then at 2000 × *g* for 20 min at 4°C. The supernatant is then centrifuged at 10,000 × *g* for 70 min at 4°C. The supernatant is further ultra-centrifuged at 100,000 × *g* for 70 min at 4°C, and the supernatant is aspirated off to obtain a residual pellet containing EVs. The pellet is re-suspended by adding 2 ml of PBS and centrifuged at 100,000 × *g* for 70 min. The supernatant is carefully aspirated off to obtain a pellet containing EV. The pellet is then re-suspended in 100–500 μl PBS and used for other downstream experiments or stored at −80°C. This method provides intact EV that can be further analyzed using nanoparticle tracking analysis (NanoSight LM10 instrument, Amesbury, UK, or similar) or examined with electron microscopy. If desired, isolation of pure exosomes or other vesicle populations can be performed by density gradient centrifugation.

### Isolation of RNA from extracellular vesicles

RNA can be isolated from EV preparations using any standard methods. We describe a protocol for isolation of RNA using TRIzol Reagent (Life Technologies, Grand Island, NY, USA). EV preparations are obtained as reported above and diluted in 100 μl PBS are incubated for 5 min at room temperature with 1 ml of TRIzol Reagent in a 1.5-ml RNase-free tube. Two hundred microliters of chloroform is added and mixed well by shaking vigorously for 30 s. The sample is then incubated for 5 min at RT and centrifuged at 12,000 × *g* for 15 min at 4°C. The upper aqueous phase is transferred to a new 1.5 ml tube without disrupting the intermediate or the bottom organic phase. Five hundred microliters of 100% isopropanol is added and incubated at −20°C for overnight. After incubation, sample is centrifuged at 12,000 × *g* for 60 min at 4°C and the supernatant removed using a pipette. Six hundred microliters of 75% ethanol is added and the sample centrifuged at 12,000 × *g* for 5 min. The ethanol is then removed and the step is repeated. The ethanol is removed using a pipette and the pellet containing RNA is air-dried prior to re-suspension in 10 μl RNase-free water. The quality of the RNA and concentration are assessed after isolation using conventional approaches such as Nanodrop 2000 (Thermo Scientific, Wilmington, DE, USA) for quantitation.

Isolations from serum samples were based on the use of SeraMir^TM^ Exosome RNA Amplification Kit (System Biosciences) after isolation of EV using Exoquick (System Biosciences). Although the SeraMir kit contains primers for PCR amplification, RNA was not amplified after isolation.

### Digital PCR for detection and quantitation of RNA

**Figure 1:**
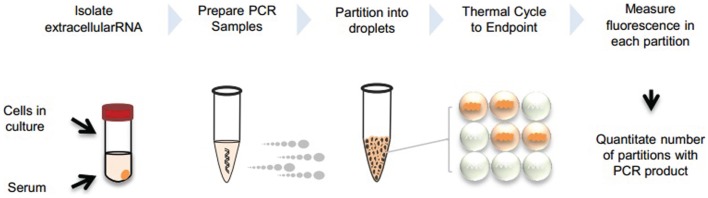
**Overview of extracellular RNA detection by dPCR**. Extracellular vesicles can be collected from cells in culture or serum. RNA is isolated from the vesicles and PCR reaction is prepared with cDNA template. Samples are partitioned into nanoliter or picoliter droplets and undergo end-point PCR. Fluorescence is measured in each partition and absolute quantitation is determined by analyzing positive and negative droplets.

We describe two approaches for dPCR analysis of extracellular RNA following reverse transcription. The first uses ddPCR (BioRad, Pleasanton, CA, USA), whereas the second uses the Raindrop (RainDance Technologies, Lexington, MA, USA). Both approaches are based on the use of partitioning into several discrete reaction volumes prior to performing PCR amplification, and followed by detection of positive reactions. An overview of extracellular RNA detection by dPCR is provided in Figure 1.

#### Droplet digital PCR analysis

Droplet digital PCR is then performed using a Bio-Rad QX100 Droplet Digital PCR system (Bio-Rad). Reactions are performed in appropriate volumes using 10 μl ddPCR 2× Master Mix, 1 μl 20× Primer and TaqMan Probe Mix, 5 μl Nuclease free water, and 4 μl reverse transcriptase product. Sample is loaded into a droplet generator cartridge. Twenty microliters of preparation sample is then transferred into the cartridge’s middle wells, being careful to avoid bubbles. Seventy microliters of oil is added into lower wells and the sample containing cartridge placed into the droplet generator to generate individual droplets. Once the process is complete, 35 μl droplets are transferred into columns of a 96-well PCR plate, sealed, and loaded into a thermal cycler. The following program is run: 95°C for 10 min, followed by 40 cycles of 94°C for 30 s and 60°C for 1 min, followed by 98°C for 10 min. After PCR is complete, the sealed plate is loaded into the droplet reader for detection of completed PCR reactions in individual droplets. The data is analyzed using the QuantaSoft software (Bio-Rad) with the thresholds for detection set manually based on results from negative control wells containing water instead of RNA.

#### RainDrop dPCR assay

Digital polymerase chain reaction reactions are prepared in 50 μl final volume, using 25 μl TaqMan Master Mix II (2×), no UNG (Life Technologies), 5 μl Droplet Stabilizer (RainDance Technologies), 2.5 μl TaqMan Assay Mix (20×), 13.5 μl nuclease free water, and 4 μl cDNA template. Droplets are generated using RainDrop Source chip (RainDance Technologies) and deposited into PCR tubes. PCR tubes are transferred into a thermal cycler and processed using the following parameters: 10 min 95°C, then 45 cycles of 95°C for 15 s and 60°C for 1 min with a ramping speed of 0.5°C/s, followed by 98°C for 10 min. After PCR completion, tubes are transferred into RainDrop Sense chip (RainDance Technologies) for fluorescence measurements. The RainDrop Analyst Software (RainDance Technologies) is used to analyze the data set to define thresholds and count droplets. Gating is performed on negative control sample and applied for all samples from the same experiment.

## Results

### Detection and quantitation of synthetic miRNA

**Figure 2:**
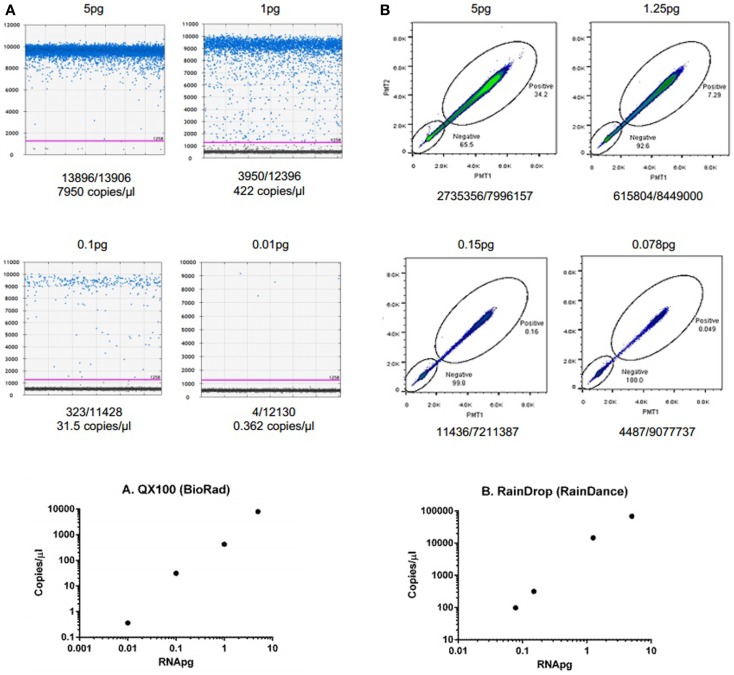
**Detection and quantitation of synthetic microRNA using dPCR**. **(A)** Fluorescence amplitude of cel-miR-39 from different concentrations of RNA using the QX100 (BioRad). cDNA was transcribed using iScript cDNA synthesis kit (BioRad, Hercules, CA, USA) in a 10-μl reaction volume. cDNA was diluted to 5, 1, 0.1, and 0.01 pg. Samples were partitioned using the Droplet Generator (Bio-Rad). PCR was performed using cel-miR-39 TaqMan Assay kit (Life Technologies). Droplets were thermal cycled to end-point and fluorescence measurement was read using Droplet Reader (Bio-Rad). Results were analyzed using QuantaSoft (Bio-Rad). **(B)** Fluorescence amplitude of cel-miR-39 from different concentrations of RNA using the RainDrop. cDNA was transcribed from cel-miR-39 using TaqMan MicroRNA cDNA synthesis kit (Life Technologies, Carlsbad, CA, USA) in a 15-μl reaction. The RT reaction was performed using a ProFlex PCR system (Life Technologies) and the following conditions: 4°C for 5 min, 16°C for 30 min, 42°C for 30 min, 85°C for 5 min, and hold at 4°C. cDNA was diluted to 5, 1.25, 0.15, and 0.078 pg dilutions. Samples were partitioned using the RainDrop Source Chip (RainDance), and PCR performed. Droplets were thermal cycled to end-point and fluorescence measurement was read using RainDrop Sense Chip (RainDance). Results were analyzed using RainDrop Analyst (RainDance). The amount of input RNA is reported in the top, whereas the number of positive/negative droplets are reported on the bottom of each panel. The bottom panels depict the relationship between calculated copies/microliter and input RNA.

To demonstrate the ability of dPCR for detection of extracellular RNA, studies were performed using an exogenous synthetic microRNA, cel-miR-39. Reverse transcription was performed using 30 ng of cel-miR-39 and cDNA generated. Various dilutions of cDNA were then analyzed using the QX100 (Bio-Rad) or RainDrop (RainDance Technologies) as described in the Section “Materials and Methods” (Figure 2). These studies demonstrate the ability of dPCR to detect input RNA amounts as low as 0.01 pg.

### Detection and quantitation of endogenous microRNA in EV and in their donor cells

**Figure 3:**
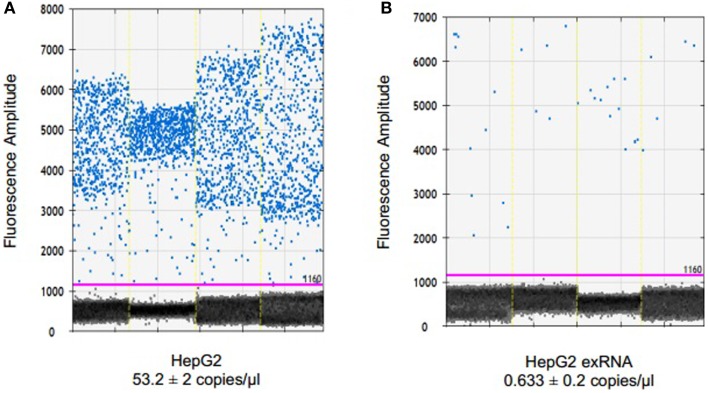
**Detection and quantitation of miR-29 in tumor cells and in EV derived from these cells**. RNA was extracted from HepG2 cells using TRIzol and from extracellular vesicles derived from these cells using ExoQuick and SeraMir (System Biosciences). For ddPCR, cDNA was generated from 132.5 ng RNA by reverse transcription. Four microliters of template cDNA was used for droplet digital PCR. Samples were partitioned using the Droplet Generator (Bio-Rad) and thermal cycled to end-point. PCR reaction was read using Droplet Reader (Bio-Rad) and results were analyzed using QuantaSoft (Bio-Rad). Fluorescence amplitude of droplets containing miR-29 in **(A)** HepG2 cells and **(B)** HepG2 EV RNA from four samples each. The average concentration of miR-29 is represented in copies/microliter, with upper and lower Poisson confidence levels.

To examine the ability of dPCR to detect endogenous non-coding RNA within EV, we examined the expression of miR-29b in HepG2 human hepatocellular cancer cells and within EV derived from these cells. miR-29 can regulate expression of MEG3, a tumor suppressive lncRNA, and miR-29 expression was down regulated in human HCC cells (14). EV RNA was isolated from HepG2 donor cells, and from EV released by these cells as described in the section “Materials and Methods.” Briefly, cells were plated in vesicle-depleted medium on 16 collagen-coated 10 cm dishes at ~70% confluency for 3–4 days. Supernatant was collected, and EV isolated using differential ultracentrifugation, and RNA isolated from EV preparations as described above. RNA isolation was also performed from donor cells using Trizol. 795.3 ng of RNA was isolated from 6985 × 10^6^ particles as quantitated by nanoparticle tracking analysis using Nanosight. cDNA was generated by reverse transcription using 132.5 ng of RNA per reaction in a 15-μl volume. ddPCR was performed using 1 ng cDNA per ddPCR reaction in a volume of 20 μl. The concentration of miR-29 in EV was 0.6 copies/μl, and was decreased compared with that in donor cells (Figure 3). Based on the measured 12 copies of miR-29 per 1 ng of cDNA, we estimate a minimum of 1.4 copies of miR-29 per 10^6^ vesicles on average. Although the expression of miR-29b expression was very low in EV, their detection was possible with ddPCR.

### Detection and quantitation of an extracellular lncRNA in human serum

**Figure 4:**
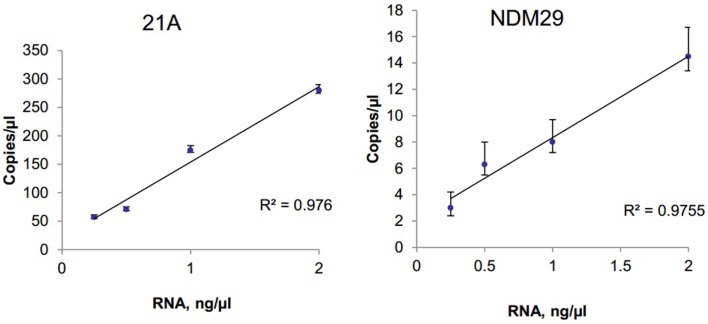
**Detection and quantitation of lncRNA in serum**. Extracellular RNA was isolated from human serum using ExoQuick and SeraMir (System Biosciences). A total of 200 ng RNA was used for RT and template cDNA was diluted to 2, 1, 0.5, and 0.25 ng for PCR. Samples were partitioned using the Droplet Generator (Bio-Rad) and thermal cycled to end-point using primers specific for lncRNA 21A or for NDM29. PCR reaction was read using Droplet Reader (Bio-Rad) and results were analyzed using QuantaSoft (Bio-Rad). The concentration of each lncRNA is expressed in copies/microliter for different amounts of input RNA with error bars representing upper and lower Poisson confidence limits (*n* = 4 replicates).

The ability to detect circulating tumor cell derived extracellular RNA is necessary to enable their use as biomarkers of disease. EV derived RNA was isolated from 1 ml of human serum using Exoquick (System Biosciences) and SeraMir™ Exosome RNA Amplification Kit (System Biosciences). RNA yield was determined using Nanodrop and a total of 183 ng RNA was obtained. cDNA was obtained using reverse transcriptase. Serial dilutions of cDNA were made and samples were analyzed using RT-qPCR or ddPCR analysis. To evaluate the expression of circulating long non-coding RNA within EV, we analyzed the expression of the long non-coding RNAs lncRNA 21A and NDM29 (Figure 4). We also analyzed expression of TUC339, a long non-coding RNA that is selectively released in EV from hepatocellular cancer cells and is foundationally implicated in modulating tumor cell behavior (15). TUC339 was not detected in EV derived from human serum by TaqMan RT-qPCR, but could be detected by ddPCR with input RNA amounts as low as 0.5 ng/μl (data not shown). Thus ddPCR may be useful as an end-point assay to detect the presence of very low expression genes that may have biological or translational potential but cannot be detected by qPCR.

## Discussion

We have described methods for measuring extracellular ncRNA expression using dPCR in isolated EV from human serum samples and from cell supernatants. Due to limited sample size and low abundance of extracellular RNA, a sensitive method of measuring gene expression such as dPCR is necessary. dPCR has an advantage for studies where the target of interest is limited or present in low quantities that cannot reliably be detected using qPCR. In addition, dPCR provides absolute quantification. The use of dPCR offers high sensitivity and enables absolute quantitation of low abundance transcripts that may be present within EV.

The ability to analyze extracellular RNA is essential for the potential application of extracellular RNA as biomarkers for early diagnosis, or as prognostic markers of disease. For human cancers, tumor-specific changes in EV RNA content could be potentially used, but such applications require sensitive and accurate determination of their RNA content. The approaches described herein may provide a dPCR based method that could be used for detecting the presence and for quantitating circulating RNA, or RNA within EV released from cells in culture. The use of dPCR could be a very useful tool for the detection and quantitation of RNA biomarkers in the clinic. In order to accomplish this, further development of dPCR based assays and standardization is required. As a first step toward this, guidelines for digital MIQE have been proposed (16). In other applications, dPCR has been shown to be able to provide precise estimates of DNA copy number with high-throughput capabilities. Therefore, we expect that further application and refinement of the approaches described will be useful for analysis of extracellular RNA as potential disease markers for human cancers and other diseases.

## Conflict of Interest Statement

The authors declare that the research was conducted in the absence of any commercial or financial relationships that could be construed as a potential conflict of interest.
